# *In vivo* characterization of the electrophysiological and astrocytic responses to a silicon neuroprobe implanted in the mouse neocortex

**DOI:** 10.1038/s41598-017-15121-1

**Published:** 2017-11-15

**Authors:** Katrien Mols, Silke Musa, Bart Nuttin, Liesbet Lagae, Vincent Bonin

**Affiliations:** 1 Neuro-Electronics Research Flanders, Kapeldreef 75, 3001 Leuven, Belgium; 2 imec, Department of Life Science Technologies, Kapeldreef 75, 3001 Leuven, Belgium; 3 KU Leuven, Department of Neurosciences, 3000 Leuven, Belgium; 4 KU Leuven, Department of Physics and Astronomy, 3001 Leuven, Belgium; 5 Vlaams Instituut voor Biotechnologie (VIB), 3001 Leuven, Belgium; 6 KU Leuven, Department of Biology, 3000 Leuven, Belgium

## Abstract

Silicon neuroprobes hold great potential for studies of large-scale neural activity and brain computer interfaces, but data on brain response in chronic implants is limited. Here we explored with ***in vivo*** cellular imaging the response to multisite silicon probes for neural recordings. We tested a chronic implant for mice consisting of a CMOS-compatible silicon probe rigidly implanted in the cortex under a cranial imaging window. Multiunit recordings of cortical neurons with the implant showed no degradation of electrophysiological signals weeks after implantation (mean spike and noise amplitudes of 186 ± 42 µV_pp_ and 16 ± 3.2 µV_rms_, respectively, n = 5 mice). Two-photon imaging through the cranial window allowed longitudinal monitoring of fluorescently-labeled astrocytes from the second week post implantation for 8 weeks (n = 3 mice). The imaging showed a local increase in astrocyte-related fluorescence that remained stable from the second to the tenth week post implantation. These results demonstrate that, in a standard electrophysiology protocol in mice, rigidly implanted silicon probes can provide good short to medium term chronic recording performance with a limited astrocyte inflammatory response. The precise factors influencing the response to silicon probe implants remain to be elucidated.

## Introduction

Neurophysiological studies^1–3^ and neural prostheses^4–6^ depend on brain implants that deliver high-quality chronic electrophysiological recordings from numerous neurons for months (rodents) and years (non-human primates, humans). Performance varies widely across devices, with some implants functioning for months to years (e.g.^7,8^) and others that fail in the first weeks post implantation^9–24^. In absence of device failure, recording quality is thought to be limited by the brain’s response to the implant^15,22,23,25–33^.

Silicon probes^34–37^ are small, scalable devices that allow high-quality extracellular recordings *in vivo* from multiple neurons simultaneously. These probes can be mass-produced at low-cost with high reproducibility and allow for CMOS integration^1,38^, on-probe processing and advanced lithographic processes enabling dense recording sites on increasingly small surfaces^2,39^. By now a mature technology, silicon probes have been widely adopted for acute recordings in small laboratory animals. However, applications in chronic implants have been lagging and their potential for brain–computer interfaces and therapeutic applications remains largely untapped^40,41^. In part at issue is a degradation in performance in terms of signal-to-noise of the recordings and overall yield over a time period of weeks, particularly in rigidly-implanted probes^10–12,14,23,25,30,31,42–46^. Though, recent attempts with modern implantation techniques have achieved excellent results^7^.

Implant-induced astrogliosis is thought to severely limit chronic recording implants^15,25–29^. Within 4 weeks of implantation, astrocytes extent their processes to encapsulate the device^22,23,26,30,31,47^. This glial scarring correlates with degradation in signal quality and neuron yield^22,23,26,30,31^. The degradation in recording quality may reflect an increased distance between the electrode and nearby neurons^22,27,32^ and/or increased impedance at the electrode–tissue interface^22,33^.

We studied *in vivo* the physiological response to a multisite silicon probe rigidly implanted in the mouse neocortex. We tested a chronic implant for mice consisting of a CMOS-compatible silicon probe implanted under a cellular imaging cranial window. Multiunit recordings of cortical neurons with the probe showed no degradation of electrophysiological signals for up to 10 weeks post implantation. Cellular imaging of fluorescently-labeled astrocytes around the probe showed a local increase in astrocyte-related fluorescence that remained stable from the second to the tenth week post implantation. These results demonstrate that, in a standard electrophysiology protocol, silicon probes can offer good short to medium-term chronic recording performance with a limited astrocyte inflammatory response. The precise factors determining chronic performance of silicon probes remain to be elucidated.

## Results

**Figure 1:**
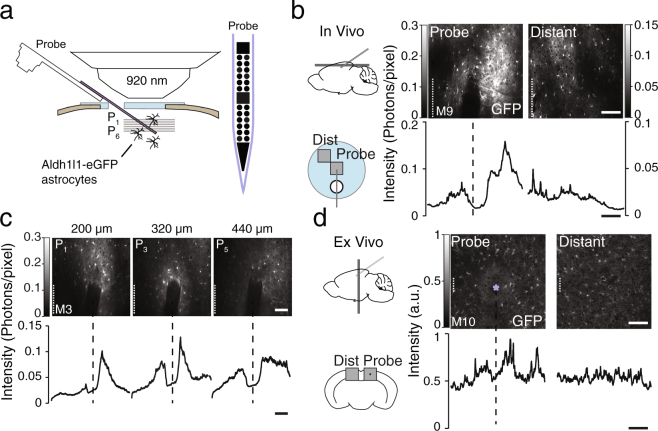
*In vivo* cellular imaging of mouse neocortical astrocytes surrounding a rigidly-implanted silicon probe. (**a**) BAC Aldh1l1-GFP^+/−^ reporter mice were implanted with a cranial imaging window^48^ and a multisite silicon probe (Supplementary Fig. S1). GFP-labeled astrocytes were imaged using a two-photon microscope (**b**) Example *in vivo* images (top) of astrocytes near the probe (left) and at a distance from it (right, ~500 µm, see inset). Images were acquired 6 weeks after implantation at a 320 µm depth below pia. Curves (bottom) show average fluorescence intensity as a function of distance from the probe over region of interest (dashed lines). Note the absence of a fluorescence increase near the probe site in the control image. (**c**) Example *in vivo* images (top) and intensity profiles (bottom) at 3 imaging depths. (**d**) Example *ex vivo* images of a coronal section centered on the probe insertion track (star symbol) (left) and at a >500 µm distance from the track (right). Note the increase in GFP fluorescence at the probe track. Scale bars indicate 100 µm.

We combined *in vivo* cellular imaging and extracellular recordings to characterize the response to a silicon probe chronically implanted in the mouse neocortex (Fig. 1a). BAC Aldh1l1-GFP^+/−^ reporter mice (background FVB/NTac, male) or Thy1-GCaMP6s-GP4.12 mice (n = 12 animals) were implanted with a fixation head plate and a cranial imaging window over the posterior cortex using a protocol known to avoid a significant neuroinflammatory response^48^. A week after head plate and window implantation, a custom-made multisite silicon probe with titanium nitride recording sites (Supplementary Fig. S1) was lowered into the cortex. The probe was inserted at a 30° angle with the horizontal through an opening in the window so that the probe’s shank lied at the window’s center. The probe base was rigidly attached to a holder, which itself was attached to the skull through the head plate (Supplementary Fig. S2a). Of the 12 animals (Table 1), 4 were implanted with dummy probes (without wire bonding) (M1, M2, M9, M10) and 8 were implanted with functional ones capable of recording (M3-M8). M9 and M10 were implanted without micromanipulator and excluded from data quantification.

**Table 1:** Summary of experimental animals.

ID	Strain	Probe	Insertion method	Purpose	Clear window	Recordings	Weeks	Reason for stopping	Note
M1	Aldh1l1-GFP	Type 1 dummy	Manipulator	Imaging	Yes	No	20	End of experiment	
M2	Aldh1l1-GFP	Type 3 dummy	Manipulator	Imaging	Yes	No	14	End of experiment	Bone regrowth
M3	Aldh1l1-GFP	Type 1	Manipulator	Both	Yes	Yes	19	End of experiment	Broken probe
M4	Aldh1l1-GFP	Type 2	Manipulator	Recording	No	Yes	8	Broken probe	Broken probe
M5	Aldh1l1-GFP	Type 2	Manipulator	Recording	No	Yes	6	Broken probe	Broken probe
M6	Thy1-GCaMP6	Type 2	Manipulator	Recording	No	Yes	27	End of experiment	
M7	Thy1-GCaMP6	Type 2	Manipulator	Recording	No	Yes	9	End of experiment	Injury unrelated to the experiment
M8	Thy1-GCaMP6	Type 2	Manipulator	Recording	Yes	Yes	25	End of experiment	
M9	Aldh1l1-GFP	Type 1 dummy	Pushed-in	Pilot	Yes	No	14	End of experiment	
M10	Aldh1l1-GFP	Type 1 dummy	Pushed-in	Pilot	No	No	4	End of experiment	No clear initial window

Dummy probes were not wire bonded. ‘Recording’ refers to electrophysiology. ‘Imaging’ refers to two-photon *in vivo* imaging.

### *In vivo* imaging of the astrocyte response to the probe

**Figure 2:**
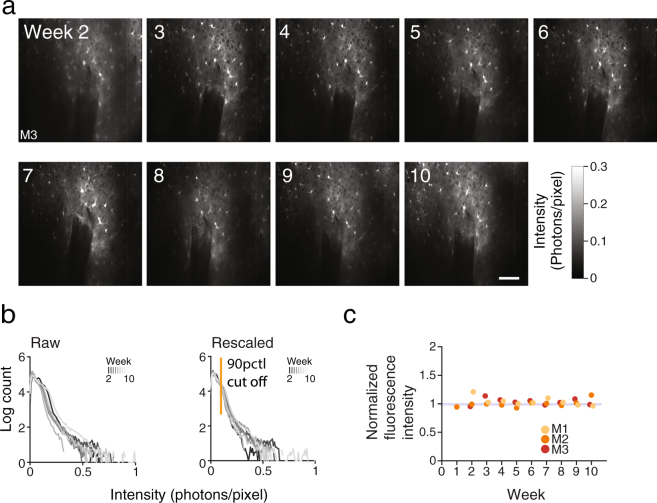
No increase in astrocyte response from week 2 to week 10 post implantation. (**a**) Time-lapse images of the same field of view for 10 consecutive weeks. Example images taken 200 µm below pia (M3). Scale bars indicate 100 µm. (**b**) Histogram of pixel intensities of images acquired in M3 on week 5 with pixel intensity cutoff (yellow) used to delineate the region of interest for calculating the average intensities reported in c. (**c**) Normalized fluorescence intensity in region of interest for each experimental session for 3 mice over 10 weeks. Fluorescence intensity normalized by median intensity from week 2 to week 10. Note how Aldh1l1-GFP expression remains stable over time.

To investigate the astrocyte response to the probe, we employed BAC Aldh1l1-GFP transgenic mice that specifically express GFP in the cytoplasm and nucleus of astrocytes in the brain and the spinal cord^49–51^. While Aldh1l1 is constitutively expressed in all astrocytes, it is upregulated in the injured cortex^51^, making it a good marker for imaging of astrocytes in proximity to and far from the injury site. The BAC Aldh1l1-GFP reporter animals showed strong GFP expression in astrocyte cell bodies and processes in both *in vivo* and *ex vivo* optical imaging (Figs 1 and 2). Immunohistochemical analysis of cortical sections cut 10 weeks after implantation showed close correspondence of Aldh1l1-GFP and the general astrocyte marker GFAP in astrocyte cell bodies around the probe track (Supplementary Fig. S3).

Two weeks after probe implantation, the cranial window provided in a subset of animals (n = 5) clear optical access to the cortex, allowing for high-resolution *in vivo* cellular imaging (Supplementary Fig. S2b). Using a two-photon microscope, we imaged the GFP-labeled astrocytes around the probe’s shank in the superficial layers of the cortex. Although the probe cast a shadow occluding the cells and processes below it, cells and processes above it and adjacent could be imaged from the surface down to a depth of 500 µm below pia (Fig. 1b, left, 1c, and Supplementary Fig. S2c).

We noted in the *in vivo* images a marked increase in Aldh1l1-GFP fluorescence in the immediate vicinity of the probe (Fig. 1b, left). This increase in astrocyte label was visible in all animals (n = 5) at multiple depths along the probe’s shank (Fig. 1c, and Supplementary Fig. S2d). Variations of this magnitude were not observed in planes parallel to the cortical surface at locations away from the probe (>500 µm within the same imaging window) (Fig. 1b, right). Post-mortem analysis of cortical sections showed a similar increase in GFP expression centered on the electrode track (Fig. 1d). Because few cell bodies were located near the probe and because GFP is expressed in the cytosol^49^, the increase in fluorescence could  reflect an increased volume of astrocytic processes near the probe.

### No increase in astrocyte response from week 2 to week 10 after implantation

To study the time course of the response, we imaged GFP-labeled astrocytes around the probe in weekly imaging sessions (Fig. 2a). In a subset of the implanted animals (n = 3), the cranial window remained free of bone regrowth so that astrocytes around the probe could be imaged at cellular resolution for 8 consecutive weeks. We acquired once a week six 500 × 500 µm images centered on the probe’s shank at depths ranging from 150 to 500 µm below the pia surface with 60 µm spacing (Fig. 1a, P_1_-P_6_, 1c, and Supplementary Fig. S2c). Recognition points such as blood vessel patterns and fluorescently-labeled cells as well as depth from pia were used to precisely target the same imaging planes in the cortex (Fig. 2a).

The pattern of labeled astrocytes around the probe was remarkably stable over time. No cell body movement and no marked change in GFP expression pattern near the probe were discerned over the duration of the observation period (Fig. 2a). To quantify the time course of the response to the probe, the images’ intensity histograms were normalized to correct for variations in fluorescence excitation across depths and across imaging sessions (Fig. 2b, see Materials). Regions-of-interest (ROIs) composed of the 10-percent brightest pixels in each fluorescence image were then examined. The ROIs generally included pixels next to the probes including labeled astrocyte cell bodies and processes at the edge of the penetrating shank and above it but excluded shadows cast by the probe and penetrating blood vessels (Supplementary Fig. S2f). We quantified the astrocyte response as the average normalized fluorescence intensity computed over each ROI and across all depths at each imaging session.

We observed in the 3 animals tested no indication of change (increase or decrease) in Aldh1l1-GFP fluorescence with time in the ROIs over the duration of the observation period (Fig. 2c, Supplementary Fig. S2e). One animal that could be imaged for 18 consecutive weeks also showed a similarly stable response (Supplementary Fig. S5c). A stable response was also observed when using a fixed threshold to select the ROIs (data not shown). This suggests that, in our preparation, in addition to the lack of cell movement or morphological changes, astrocyte response occurs rapidly and remains stable in the first weeks post implantation.

**Figure 3:**
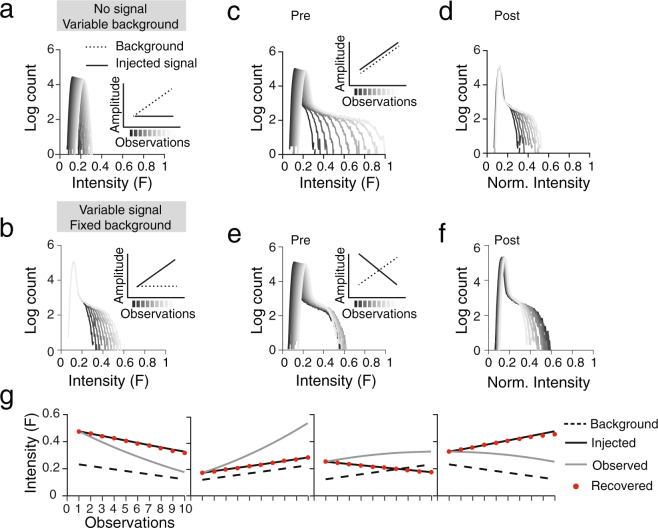
Application of image analysis methods to a synthetic data set. (**a–b**) We generated data consisting of a low-intensity background (**a**) and a high-intensity target (injected signal) (**b**)  to simulate the fluorescence intensity and laser power differences found in the experimental data. Simulated data consisted of a set of random numbers from the Poisson distribution with mean lambda (**a**), multiplied with a Gaussian blob with varying intensity (**b**). (**c**–**f**) Pixel intensity histograms before (**c**,**e)** and after (**d**,**f**) correction to allow for comparison of GFP expression across weeks. Images were rescaled by matching the pixel values between the 0^th^ and 90^th^ percentile (dark pixels) in the intensity histogram. Examples show the cases of (**c**,**d**) a simultaneously increasing background and injected signal and (**e**,**f**) a decreasing injected signal on an increasing background. (**g**) After correction, the rescaling method was capable of recovering the injected signal regardless of an increase or decrease in input signal or laser power. Each data point is the mean signal intensity of the bright pixels (>90^th^ percentile), simulating the region of astrocyte reactivity.

To demonstrate that the image normalization procedure did not bias the quantification of the results, we applied the same analysis to a set of synthetic images (Fig. 3), consisting of a low-intensity background (Fig. 3a) and a high-intensity target (injected signal) (Fig. 3b). We considered four scenarios in which illumination and injected signal were varied independently, or were varied together in the same or opposite directions (Fig. 3c–f). The analysis faithfully recovered the injected signal in the synthetic images across all scenarios (Fig. 3g).

We conclude that, with a standard electrophysiological protocol, the local astrocytic response to a rigidly implanted multisite silicon probe is limited and shows little change from the second week up to the tenth week after probe implantation.

### No degradation in signal quality for 10 weeks after implantation

**Figure 4:**
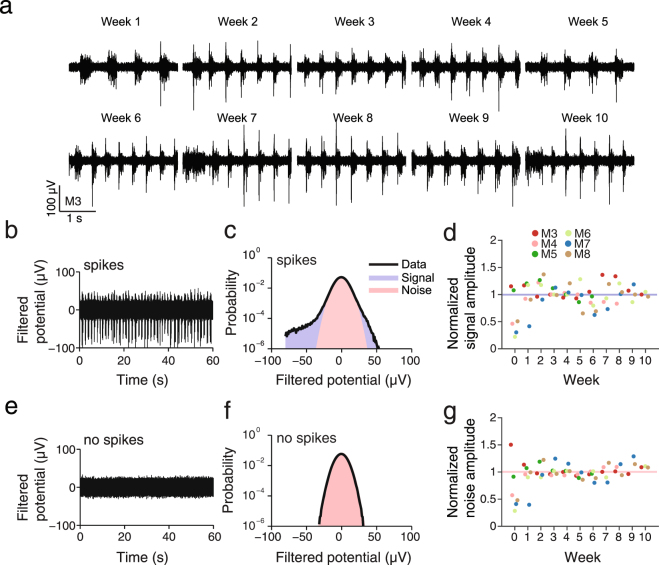
No degradation in signal quality for up to 10 weeks after implantation. (**a**) Example recordings from the probe in M3 from 1 recording site for 10 consecutive weeks. Multi-unit activity was observed for each week. (**b**) Extracellular recording from a channel with multiunit activity and (**c**) corresponding logarithmic histogram with estimates of signal (red) and noise (blue). (**d**) Normalized signal amplitude for 6 mice over 10 weeks. The amplitude was normalized by the median amplitude from week 2 to week 10. Note how the signal amplitude remains stable over time. (**e**) Extracellular recording from channel without multiunit activity and (**f**) corresponding logarithmic histogram. The standard deviation of the normal distribution was calculated as an approximation for the noise. (**g**) Normalized noise amplitude for 6 mice over 10 weeks. The amplitude was normalized by the median amplitude from week 2 to week 10. Note how the noise amplitude remains stable over time.

To investigate whether the observed lack of change in astrocytic response is reflected in the quality of neural recordings, we examined the electrical signals from the chronically-implanted silicon probes (Fig. 4 and Supplementary Fig. S4). We recorded extracellular signals from the probes in weekly sessions over a ten week observation period (Fig. 4a) (n = 6 animals) and quantified the measured spontaneous multiunit activity (Fig. 4b,c,e,f). The recordings were made at room illumination (ambient light) while animals were head fixed under light ketamine anesthesia (n = 3 animals) or in the quiet wakeful state (n = 3 animals).

We observed in all animals tested robust multiunit activity from the probes through the ten week observation period (Fig. 4a). We quantified multiunit activity with measures of signal and noise amplitude by examining the extracellular potential histograms. We estimated noise by fitting a Gaussian density function to the extracellular potentials, excluding values above 3 standard deviations (Fig. 4e,f). We estimated signal by subtracting the fitted Gaussian histogram of the noise from the extracellular potential histogram. We defined signal amplitude as the difference between 99^th^ and the 1^st^ percentiles of the result (Fig. 4b,c). This measure of signal estimates  the peak-to-peak amplitude of the largest action potentials observed on the channels whereas the noise measure reflects the combined effects of thermal noise and background neural activity.

Beyond a settling period of 1 to 2 weeks post implantation, the recordings showed no signs of degradation in signal amplitude nor an increase in noise amplitude for 10 weeks post implantation (Fig. 4d,f). Across animals, the mean signal amplitude was 186 µV peak-to-peak for 5 animals with probes with 10 µm contacts (Fig. 4d and Supplementary Fig. S4c) (SD = 41.8 µV, n = 5 animals), and 80 µV for 1 animal with a probe with 25 µm contacts. All measures were calculated over a ten week period after implantation. The mean noise amplitude over that period of time was 16 µV_rms_ for 10 µm contacts (SD = 3.2 µV, n = 5 animals) and 6.0 µV_rms_ for 25 µm contacts (n = 1 animal) (Fig. 4d and Supplementary Fig. S4d), corresponding to average peak signal-to-noise ratios of 2.5 and 2.2, respectively (Fig. 4f). We obtained similar results in a subset of animals that were monitored for 18-28 weeks post implantation (Supplementary Fig. S5) (n = 3 animals).

We conclude that, with a standard electrophysiological protocol, we do not observe an increase in noise amplitude or a degradation in signal amplitude of extracellular multiunit recordings with a chronically-implanted multisite silicon probe, for at least 10 weeks post-implantation.

## Discussion

We studied *in vivo* the response to a multisite silicon probe rigidly implanted in the mouse neocortex. We tested a lightweight implant made of a CMOS-compatible titanium nitride silicon probe and a cranial window for cellular imaging. Multiunit activity was stable for weeks after implantation with no indication of signal degradation (n = 5 mice). Two-photon imaging through the cranial window in BAC Aldh1l1-GFP reporter mice enabled visualization of fluorescently labeled cortical astrocytes around the probe over a period of days to months (n = 3 mice). The imaging showed a local increase in astrocyte-related fluorescence that remained stable from the second to the tenth week post implantation. This suggests that silicon probes can provide good short to medium term chronic recording performance with a limited astrocyte inflammatory response. The lack of excessive inflammation and the absence of recording quality decline we observe suggest the titanium nitride electrode site material may be suitable for chronic applications. While we demonstrated good short and medium performance, long-term performance still needs to be investigated.

While modest, the imaging results are amongst the first *in vivo* longitudinal characterization of the astrocytic response to a chronically implanted probe. Most data on tissue response to chronic implant stems from static endpoint methods^26,27,52,53^ which cannot reveal response evolution in individual animals. Two-photon microscopy was successfully applied to study the immediate microglia response to probe insertion^53^ as well as its impact on the blood brain barrier^54^, and to study the chronic astrocyte response to acute injury^55^. A limitation of the approach is the requirement of a functional electrophysiological implant while maintaining optical access to the brain through the observation period. Brain hemorrhage and edema following probe insertion can severely impair the *in vivo* cellular imaging (Supplementary Fig. S2b). This complicates investigation of the initial cellular response to injury and could prevent chronic investigations of more severe injuries. For the former question, acute imaging experiments may be more appropriate^53^. Additionally, the presence of the probe makes it difficult to remove the glass window to deal with bone regrowth, also detrimental to cellular imaging^48^. This could be alleviated by replacing the glass window with a more flexible substrate. Together, these factors limit the success rate of the experiments and make it a challenge to obtain good optical and electrical measurements from the same animal. For this reason, a correlation between the *in vivo* response and the electrical recordings, still remains to be demonstrated. The cellular imaging approach, however, could be useful by itself in testing, for example, new probe materials and coatings and for examining the *in vivo* behavior of various cell types.

The local increase in Aldh1l1-GFP fluorescence *in vivo* near the probe could reflect a response to the probe. There was a good agreement between Aldh1l1-GFP and the standard neuroinflammatory marker GFAP (Supplementary Fig. S3). The increase in fluorescence could reflect increases in Aldh1l1-GFP expression, or an increase in the volume of extracellular processes because GFP is targeted to the cytosol.  While we used Aldh1l1, the approach would be amenable to other markers. For astrocytic markers, Aldh1l1 has the advantage that it is distributed over the entire cell, including finer structures, whereas GFAP is localized mainly to somas and larger processes^51^. The lack of increase in Aldh1l1-GFP expression suggests the response reached an asymptote within 2 weeks of implantation. As in Bardehle and colleagues^55^, we have seen little indication of astrocyte cell body movement.

We observed little degradation of the quality of multiunit recordings over the ten week observation period. Both signal and noise amplitudes showed no large variations from two weeks after implantation and onward. The observed stability in this work may stem from the rigidity of the preparation. Early applications of silicon probes have frequently observed degradation over time^12–14^ which has limited applications in chronic implants. A workaround has been to mount the probe on a microdrive and move the probe to keep good signals^37^. However, a recent study with rigidly implanted probes has shown good short to medium term performance without microdrives^7^ which suggests other factors are at play. A possible factor that might have contributed to the good performance of the probe in our study is the glass window which stabilizes the cortex^48^ and possibly reduces probe movement relative to the brain. Repetitive brain motion relative to the probe may result in mechanical injury which can foster inflammation^22,56^. Alternative solutions are un-tethered probes^52,57^ and probes fabricated in a compliant material^58–60^ or with low density^61^, which have all been shown to reduce inflammatory responses.

## Materials and Methods

### Animals and cranial window implantation

Animals were handled in accordance with international (EU Directive 86/609/EEC) and national laws governing the protection of animals used for experimental purposes, minimizing distress during procedures. All procedures were approved by the Ethical Committee for Animal Welfare (ECD, Ethische commissie Dierenwelzijn) of KU Leuven and imec. Nine BAC Aldh1l1-GFP^+/-^ reporter mice (M1-M5, M9-M10) and three Thy1-GCaMP6s-GP4.12 (M6-M8)^62^ adult male mice (3 to 6-month-old and weighing 30–40 g) were implanted with a perforated cranial glass window and  a silicon probe for neural recordings (Table 1)^48^.

The mice were anesthetized with isoflurane (induction 3%, 0.8 L/min O_2_; maintenance 1–1.5%, 0.5 L/min O_2_), mounted on a stereotaxic apparatus and kept at physiological temperature by a homeothermic blanket; the eyes were protected with antibacterial ointment (Duratears, Alcon). The scalp was shaved, disinfected (70% ethanol and Betadine), opened and removed to expose the cranium. The periosteum was removed and the lateral and posterior muscles were retracted. Vetbond (Vetbond, WPI) was applied to open skin and exposed muscle. A custom-designed probe holder (Supplementary Fig. S2a) and titanium head plate (Supplementary Fig. S2a) were positioned over the left posterior cortex (1.25 mm anterior to lambda, 3.1 mm lateral to midline) and attached to the skull using cyanoacrylate glue, super-bond (Super-bond, Generique international) and dental cement (Kerr Tab, Kerr Dental). A 5 mm craniotomy was performed in the center of the head plate and a perforated (1 mm opening) translucent glass window made of one 8-mm and two 5-mm concentric cover slips (Harvard Apparatus) glued with optical adhesive (Norland Products) was placed on the brain. The cranial window was affixed with vetbond and dental cement mixed with black tempera pigment to provide light shielding during fluorescence imaging. The cranial window aperture was filled with low-toxicity silicon sealant (Kwik-Sil, WPI). Mice received 72 h post-operative care and recovered for 5 days.

### Silicon probe implantation

After the recovery period, the mice were habituated to head fixation in 3 sessions. During each session, the animal was first allowed to explore the setup and was then head fixed for an increasing period of time (10, 30, 60 minutes). Mice were monitored for signs of distress.

About one week post implantation, a custom-made multisite silicon probe (imec, Leuven, Belgium)^38,63,64^ was attached to the holder and inserted in the animal’s posterior cortex through the aperture in the cranial window. The probes had a single shank of 10-mm length, 100-µm width, and 50-µm thickness except for M2, which had a probe with a 200-µm wide shank (‘Type 3”). The probes bore 24 (“Type 2”) or 29 (“Type 1”) titanium nitride recording sites of 10 or 25-µm diameters, respectively (Supplementary Fig. S1). The impedance of the 10 or 25 µm recording sites, measured with a nano Z device (Neuralynx), was 409 ± 27 kΩ and 67 ± 18 kΩ (mean ± SD for n = 24 or 29 recording sites, 1 kHz in saline), respectively (Supplementary Fig. S4a,b). The animal was head fixed and anesthetized with a ketamine-medetomidine mixture (Anesketin, Eurovet Brussels, 7.5 mg/ml) (Domitor, Orion Pharma, 0.1 mg/ml) (7.5% ketamine and 10% medetomidine in 0.9% saline) (7 µl/g). The window was disinfected with 70% ethanol, the silicon sealant removed and a slit in the exposed dura was made through the aperture in the cranial window. The silicon probe was mounted on a micromanipulator and inserted at a 30° angle into the holder until the probe tip reached the brain surface. The probe was then lowered into the cortex at a rate of 10 µm/s down to a depth of 500 µm from pia (1 mm insertion). After 20-30 minutes of recovery, the probe was lowered further until stable spiking activity was observed. The final depth of the probe tip was 500 – 800 µm (1.6 mm insertion) from pia, corresponding to 350-650 µm depth from pia for the active recording area. The probe was cemented in place to the support structure and the exposed tissue in the window opening around the probe was covered with silicon sealant. Anesthesia was interrupted with atipamezole (Antisedan, Orion Pharma, Finland) and animals were let to recover for 2 days.

To enable longitudinal, cellular-resolution imaging of astrocytes near the probe and to limit the potential influence of the cranial window^48^ on the results, the probe was inserted one week after cranial window implantation in a region deemed free of blood vessels using an optical microscope. The two-photon imaging was also performed at several hundred microns away from the insertion site. These two measures which were needed for imaging may have contributed to the limited inflammatory response to the probe we observed.

### Extracellular recordings and signal-to-noise analysis

The first recording session took place on the day of probe implantation (“week 0”). Subsequent sessions (“week 1” and up) were performed at seven day intervals for up to 28 weeks. Animals were awake (M6-8) or lightly anesthetized (ketamine-medetomidine mix, 5 µl/g) (M3-M5) and head fixed. Extracellular signals were recorded at ambient illumination on a portable USB-ME32-FAI-System (Multi Channel Systems) at 25,000 samples/s, band-pass filtered at 300 to 5000 Hz and analyzed with MATLAB (The MathWorks, Boston, MA). To subtract common noise, the recording system’s ground was attached to the animal’s head plate and the top right recording site was used as internal reference. Data from malfunctioning electrodes (standard deviation of the filtered signal is smaller than 2 µV) and reference channels were discarded and data from the remaining channels were concatenated. The ‘noise’ amplitude was estimated by fitting a Gaussian density function to the extra-cellular potential histogram of the concatenated data, excluding values above 3 standard deviations. The ‘signal’ amplitude was estimated by subtracting the fitted noise histogram from the extracellular potential histogram and computing the difference between the 99^th^ and 1^st^ percentile of the resulting histograms. The signal-to-noise ratio was calculated as the ratio of signal peak-to-peak amplitude over 6 times noise standard deviation. To examine variations of noise and signal across weeks, measurements were normalized by the median signal and noise between week 2 and the last week. To confirm the integrity of recording sites and head-plate ground, weekly *in vivo* ‘impedance’ measurements between probe sites and the head plate were performed from Week 1 and onward. For the probes with 10 and 25 µm recording sites, *in vivo* ‘impedance’ was 3.44 ± 0.84 MΩ and 2.67 ± 0.27 MΩ (mean of 6 probes ± SD for n = 24 or 29 recording sites, 1 kHz), respectively.

### Imaging acquisition and analysis

Imaging experiments were performed once a week for 10 weeks. Animals were head-fixed and lightly anesthetized with isoflurane (<1% in air) or imaged awake. In each session, 6 planes were imaged with a resonant scanner two-photon microscope (Neurolabware LLC), a 16x lens (Nikon, CFI75 LWD 16xW, NA 0.80, working distance 3 mm) and a Spectra-Physics Mai Tai HP DeepSee tunable ultrafast infrared laser at depths between 150/200 to 500 µm (steps of 60 µm) with laser powers ranging between 20 and 120 mW at 920 nm. For each imaging plane, 2048 frames (720 by 962 pixels) were imaged at a frame rate of 15 Hz. Each week, the imaging location was determined by visually matching the position of cells and vessels on the image of the first week.

All imaging data was analyzed with MATLAB (The MathWorks, Boston, MA). The imaged frames were first corrected for drift and motion by using rigid registration (TurboReg, ImageJ), and subsequently averaged. The averages were subdivided into low and high intensity subregions (threshold 90^th^ percentile). Images were rescaled to match the histograms of the low intensity subregions (least square distance). The average pixel intensity of the high intensity subregions was used to quantify astrocytic response.

The validity of the analysis method was tested by applying it to a set of simulated data, consisting of the multiplication of a low intensity background and high intensity target (injected signal), all generated in MATLAB (see Fig. 3). Background was simulated as a Poisson distribution with varying mean; the injected signal was a Gaussian blob with varying peak intensity. Both signal and background were varied for the following conditions: 1) decreasing signal and decreasing background, 2) increasing signal and increasing background, 3) decreasing signal and increasing background, 4) increasing signal and decreasing background.

### Histology

At the end of the experiment, mice were euthanized with a ketamine overdose (Anesketin, Eurovet Brussels) (200 mg/kg) and transcardially perfused with 1X Phosphate Buffered Saline (PBS), followed by 4% paraformaldehyde in distilled water. Brains were collected and kept in 4% formaldehyde in distilled water overnight, transferred to PBS with 20% sucrose, and preserved at 4 °C. The frozen brains were cut in 20 μm thick coronal slices with a cryostat. The slices spanning the lesion site were selected and transferred to glass slides. Tissue slices were prepared for immunohistochemistry by 2 h treatment in 20% normal goat serum with 0.2% Triton X-100 in PBS after a rehydration step for 15 min in PBS. After washing with PBS, the slices were overnight incubated with primary antibodies at room temperature (Chicken, anti-GFAP, 1:500, Millipore). After washing with PBS, secondary antibodies were incubated for 2 h (Goat, anti-chicken Alexa Fluor 647, 1:500, Molecular Probes). After washing with PBS, sections were mounted with Vectashield containing DAPI (Vector Laboratories) and sealed with nail polish.

### Data

Data will be made available upon request to the author.

## Electronic supplementary material

Supplementary Figures
