# Supplementary material for: In vivo characterization of the electrophysiological and astrocytic responses to a silicon neuroprobe implanted in the mouse neocortex
Source: Sci Rep. 2017 Nov 15;7:15642. doi: 10.1038/s41598-017-15121-1 (PMC5688150; doi:10.1038/s41598-017-15121-1)
Supplement: Supplementary file 1 — Supplementary Figures [file 41598_2017_15121_MOESM1_ESM.pdf]

Supplementary Information

In vivo characterization of the electrophysiological and astrocytic responses to a silicon neuroprobe implanted in the mouse neocortex

Katrien Mols[1,2,3], Silke Musa[2], Bart Nuttin[3], Liesbet Lagae[2,4], Vincent Bonin[1,5,6]
[1]Neuro-Electronics Research Flanders, Kapeldreef 75, 3001 Leuven, Belgium.
[2]imec, Life Science Technologies Department, Kapeldreef 75, 3001 Leuven, Belgium.
[3]KU Leuven, Department of Neurosciences, O&N II Herestraat 49 - box 721, 3000 Leuven, Belgium.
[4]KU Leuven, Department of Physics and Astronomy, Celestijnenlaan 200d - box 2412, 3001 Leuven, Belgium.
[5]VIB, 3001 Leuven, Belgium.
[6]KU Leuven, Department of Biology, Naamsestraat 59, 3000 Leuven, Belgium.
Correspondence to: vincent.bonin@nerf.be

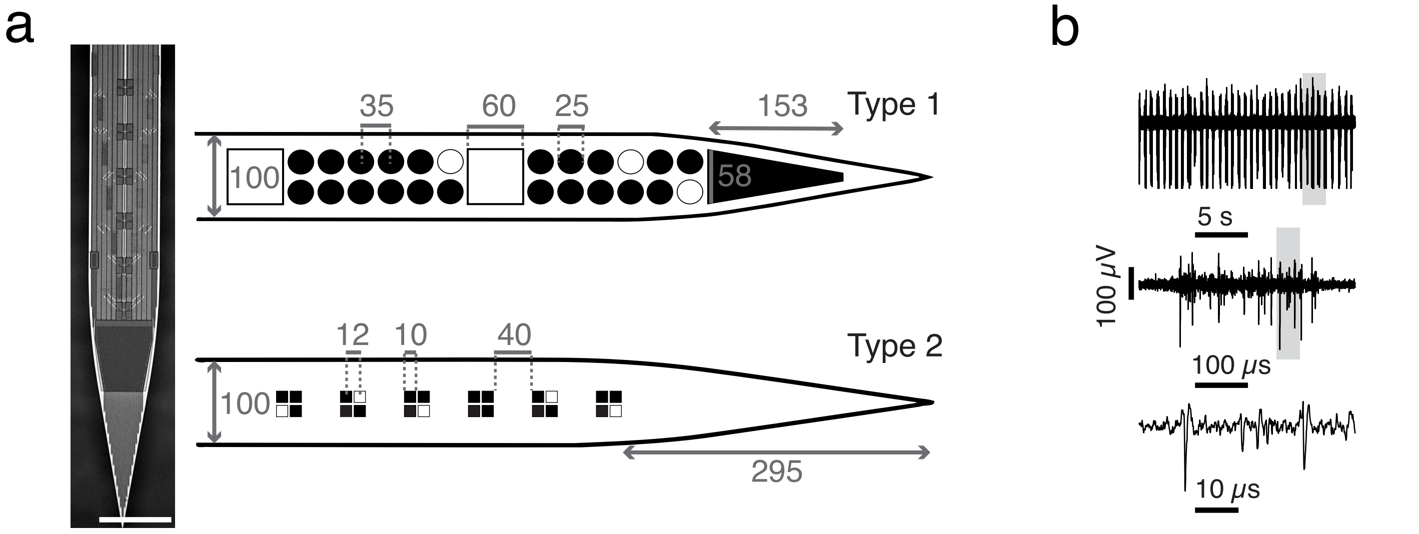

Supplementary Fig. S1. Multisite silicon probes with titanium nitride recording sites (a) Probe layout. Confocal image of Type 2 probe (left) and dimensions per probe type (Right). All probes are 10 mm long and 50 μm thick, with titanium nitride as contact material and silicon as carrier. M3, M9 and M10 were implanted with Type 1 probes, M2 with a 200 μm wide dummy probe (Type 3, not shown) and M4-M8 with Type 2 probes. The scalebar indicates 100 μm. (b) Example recordings from M3, week 3. Imec's silicon probes are suited for detecting high-quality multi-unit recordings.

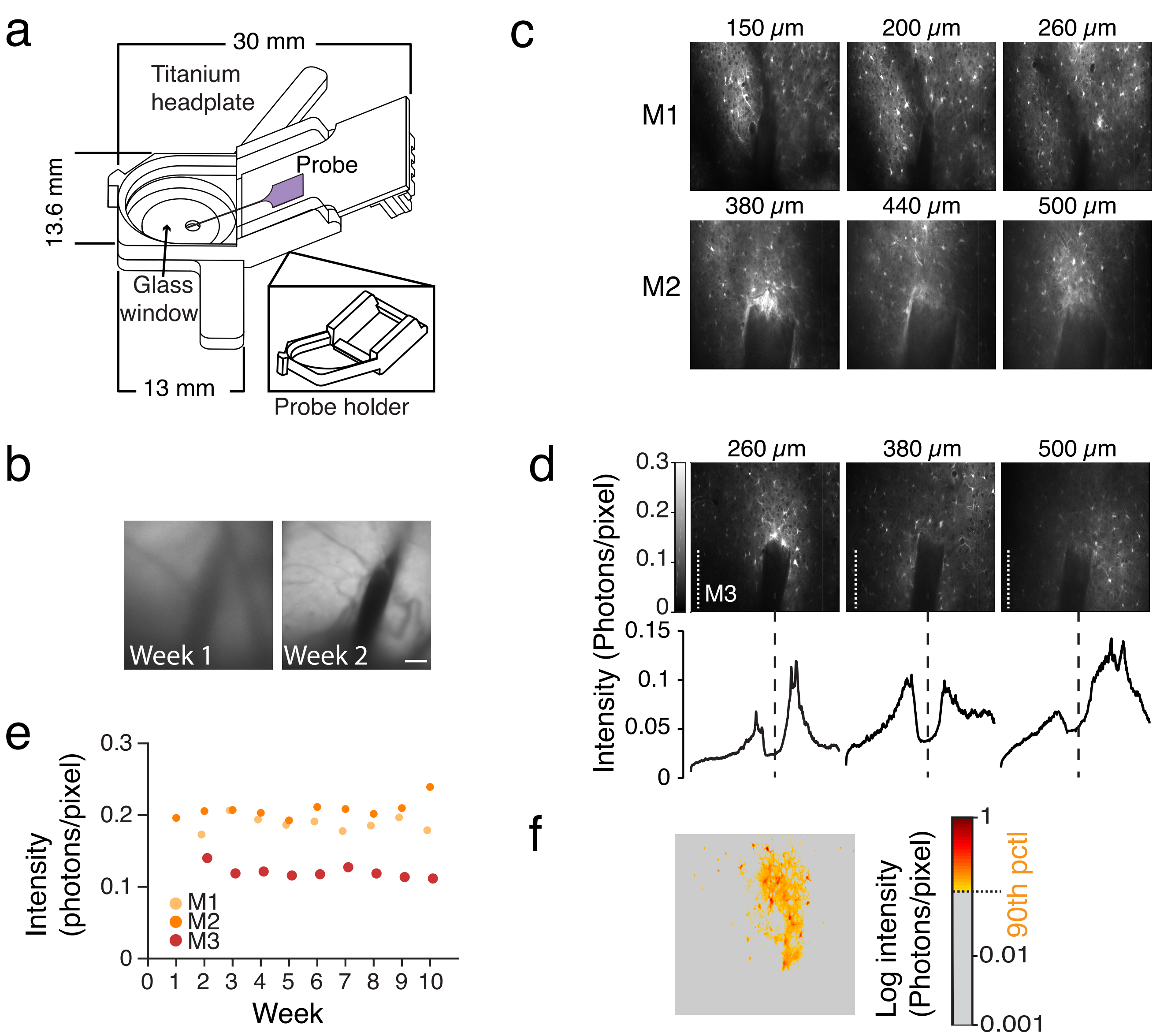

Supplementary Fig. S2. Chronic cranial window preparation for imaging the cellular environment around the probe. (a) Head mount for cellular imaging around a chronically implanted probe. Probes were implanted through an opening in the glass window and chronically held in place by a 3D- printed probe holder (inset). (b) One-photon image of the surface of the brain around the probe. After 1 to 2 weeks residues from the probe implantation have cleared out and the window is ready to be imaged. (c) Example images from 2 mice over 3 different depths. (d) Example images at 3 depths (top) and corresponding intensity profiles (bottom). Note the increased fluorescence intensity next to the probe. Intensity profiles were obtained by averaging over the region between the dashed lines. (e) Average fluorescence intensity of the 10% brightest pixels as a measure for the astrocyte response. Scale bars indicate 100 μm. (f) Log intensity image of the 10% brightest pixels in the images defining a region of interest, which is used for further analysis as a measure of astrocyte response. The region of interest consisted of labeled astrocyte cell bodies and processes at the edge and above the probe, but excluded shadows cast by the probe and the blood vessels. Example from M3, week 6, at 200 μm below pia.

a

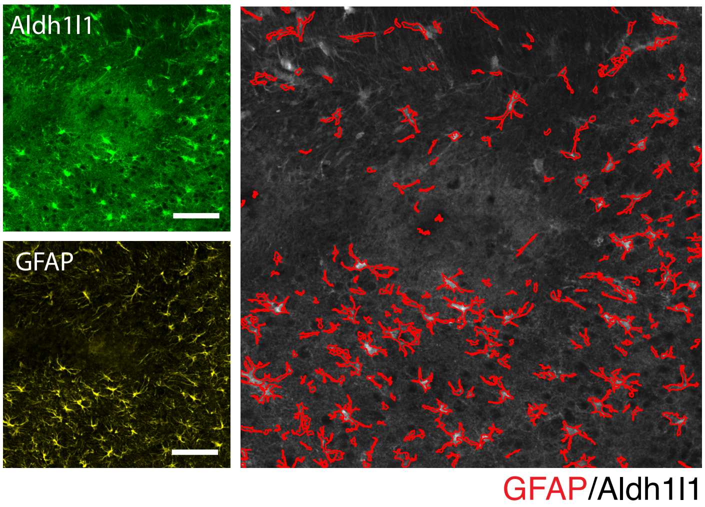

Supplementary Fig. S3. Correspondence of Aldh1l1 and the standard immunohistochemistry marker GFAP. Ex vivo data showing intrinsic fluorescence of Aldh1l1-GFP (top left) and the labeled GFAP-protein (bottom left). The overlay (right) shows that all GFAP-cells are also expressing Aldh1l1. Scalebars indicate 100 μm.

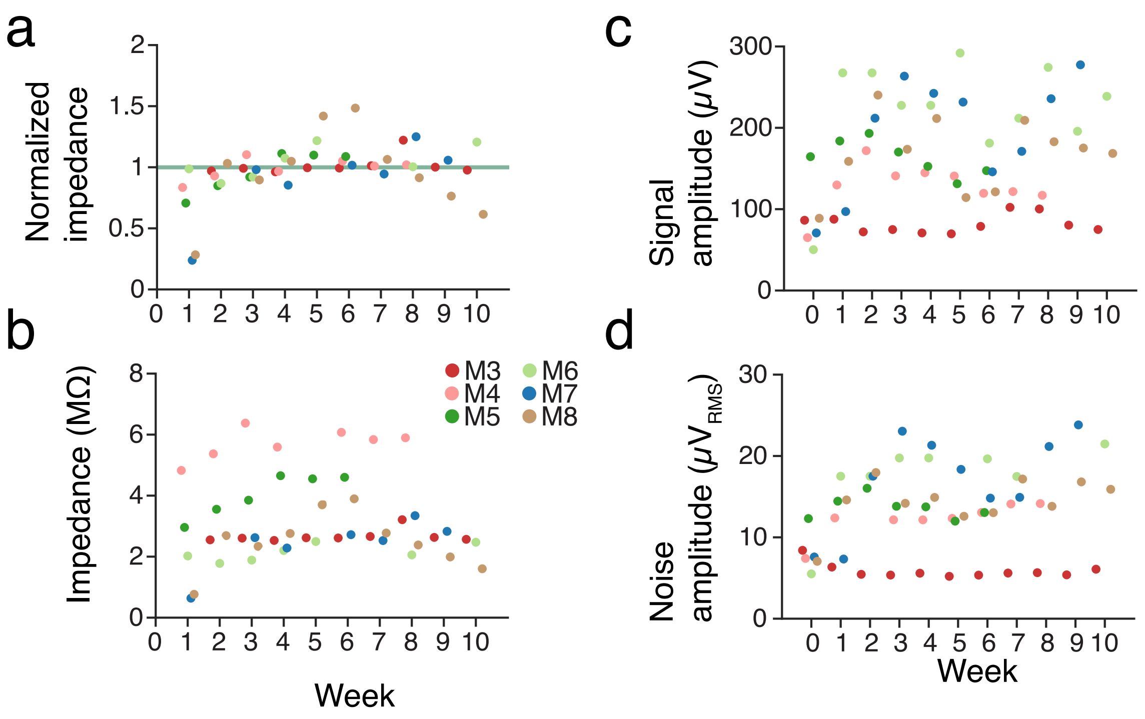

Supplementary Fig. S4. No degradation in probe performance for 10 weeks after implantation. (a) Median impedance normalized by the median over the measurements. Note the stable trend over the experimental weeks. (b) Raw impedance data. The average impedance was 3.24 MOhm or 2.70 MOhm for 10 μm and 25 μm contacts respectively. In all cases it was sufficiently low to pick up small amplitude spikes. (c) Mean signal amplitude per mouse. (d) Median noise floor for all mice.

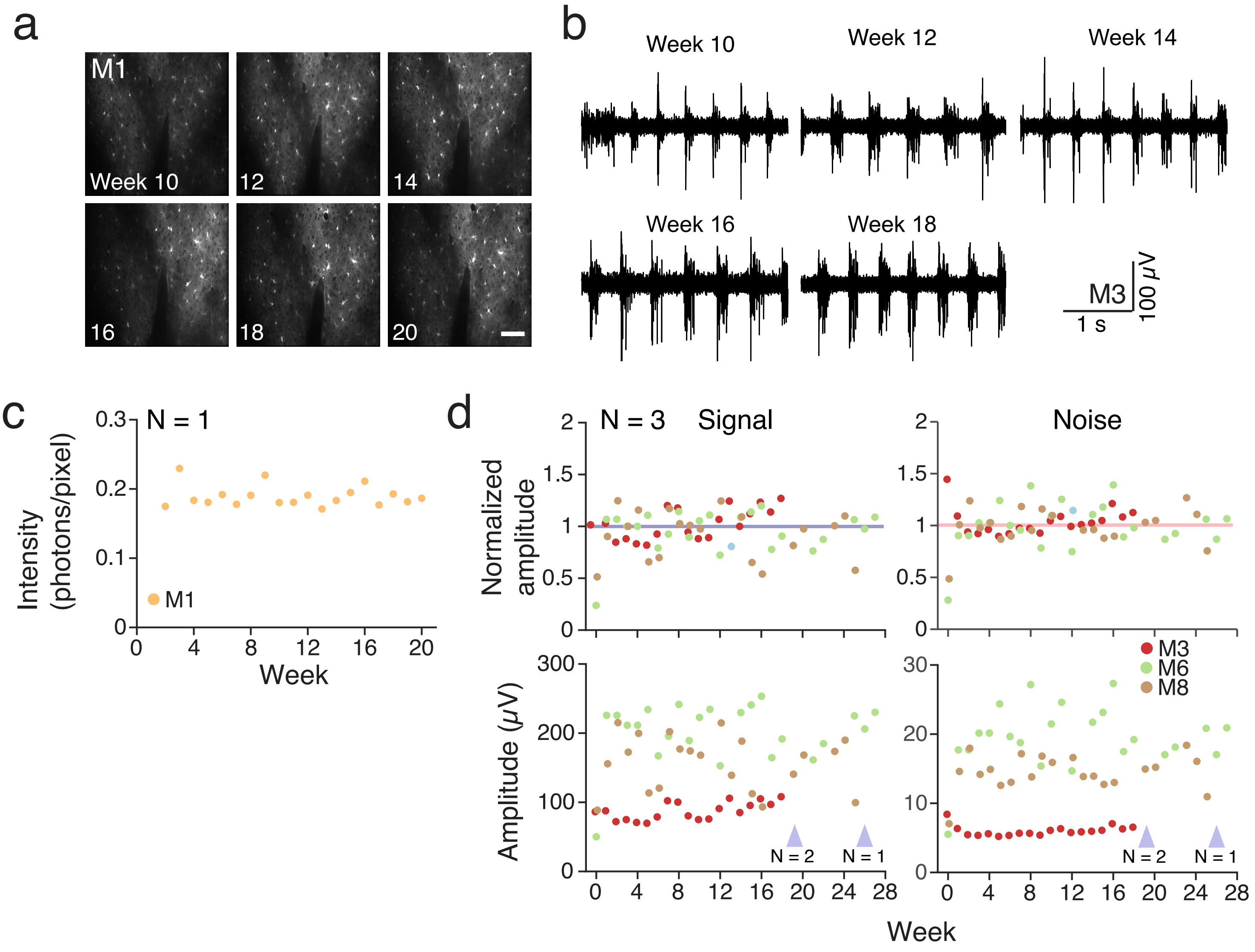

Supplementary Fig. S5. Example data suggest no signal quality decline beyond 10 weeks. (a) Imaging examples from one case study (M1). The same field of view was imaged up to 20 weeks. (b) Example recordings from M3 up to 18 weeks. (c) Average fluorescence intensity of the 10% brightest pixels representing the astrocyte response. A stable trend was visible up to 20 weeks post-implantation. (d) Average peak-to-peak multi-unit activity and noise from a subset of animals (N = 3). The top shows values normalized to the median value from week 2 to the end week; the bottom shows raw values. We did not observe any signal disappearance up to 27 weeks post-implantation.
